# Is It Homœopathy or Isopathy?

**Published:** 1892-02

**Authors:** Samuel Swan


### IS IT HOMŒOPATHY OR ISOPATHY?

Editor of The Homœopathic Physician:

I do not intend to say that the potentization of a morbific virus makes it homœopathic to any and every disease whatever, but I should have stated more clearly that it would be homœopathic, as Hahnemann says, to the "*identical virus*" with which the organism is tainted. The virus that causes a specific disease—for instance, malarial fever—is an entity, but is made to appear as different diseases by the personality of the patient. There are a number, perhaps all zymotic diseases, which are brought into existence by blood-poison; hence in the initiative stages Pyrogen will be found to modify the condition by curing the blood-poison, and often will effect an entire cure, especially if no other latent miasm is awakened. To a greater or less extent the access of measles, scarlet fever, small-pox, typhus fever, yellow fever, malarial fever, and blood-poison are similar. In all are found the initial chill, fever, pains in the lower extremities, backache, headache, nausea, vomiting, and a feeling of prostration; sore throat, general malaise, and often delirium and convulsions.

If an exhaustive proving of Pyrogen were made it is probable that the symptoms of all of the diseases would appear. There are plenty of young men and women who would make the proving if they were thoroughly informed concerning it. In reading the splendid proving of Lac-caninum by Dr. Morgan—the symptoms so significant of diphtheria and which have rendered it the most valuable remedy in that disease, never kept her in the house a day. If any willing to prove Pyrogen will confer with me, I will give them the best potency and full advice for their guidance.

Samuel Swan.

---

Foot Note.—See The Homœopathic Physician for November, 1891, page 425, and December, pages 443, 444.

